# Supplementary material for: Comparative genomics provides new insights into the diversity, physiology, and sexuality of the only industrially exploited tremellomycete: Phaffia rhodozyma
Source: BMC Genomics. 2016 Nov 9;17:901. doi: 10.1186/s12864-016-3244-7 (PMC5103461; doi:10.1186/s12864-016-3244-7)
Supplement: Additional file 6: — List of orphan genes with links to PFAM (related to Additional file 1: Table S1). (ZIP 1428 kb) [file 12864_2016_3244_MOESM6_ESM.zip › PFAM_UNIQUE/G03462_P.html]

#                                                                            --- full sequence --- -------------- this domain -------------   hmm coord   ali coord   env coord
  
# target name        accession   tlen query name           accession   qlen   E-value  score  bias   #  of  c-Evalue  i-Evalue  score  bias  from    to  from    to  from    to  acc description of target
  
#------------------- ---------- ----- -------------------- ---------- ----- --------- ------ ----- --- --- --------- --------- ------ ----- ----- ----- ----- ----- ----- ----- ---- ---------------------
  
G03462\_P             -            366 Shisa                PF13908.1    179       1.1    8.1   0.0   1   3      0.65   6.5e+02   -0.9   1.3   115   150    87   124    77   175 0.56 - Wnt and FGF inhibitory regulator  
G03462\_P             -            366 Shisa                PF13908.1    179       1.1    8.1   0.0   2   3    0.0011       1.1    8.1   0.0    67   115   167   213   142   237 0.76 - Wnt and FGF inhibitory regulator  
G03462\_P             -            366 Shisa                PF13908.1    179       1.1    8.1   0.0   3   3       0.3     3e+02    0.1   0.7   119   161   300   320   261   343 0.52 - Wnt and FGF inhibitory regulator
